# The Role of Systematic Lymphadenectomy in Low-Grade Serous Ovarian Cancer: A Systematic Review and Meta-Analysis

**DOI:** 10.3390/cancers16050955

**Published:** 2024-02-27

**Authors:** Rosa Montero-Macías, Juan José Segura-Sampedro, Pascal Rigolet, Fabrice Lecuru, Andrea Craus-Miguel, Juan Manuel Castillo-Tuñón

**Affiliations:** 1 Department of Gynecology and Obstetrics, Hospital Center of Poissy Saint Germain en Laye, 78300 Poissy, France; 2 Section of Peritoneal, Retroperitoneal and Soft Tissue Oncological Surgery, General & Digestive Surgery Service, La Paz University Hospital, IdiPAZ, 28046 Madrid, Spain; 3 School of Medicine, University of the Balearic Island, 07122 Palma de Mallorca, Spain; 4 Health Research Institute of the Balearic Islands (IdISBa), 07009 Palma de Mallorca, Spain; 5 Curie Institute, Paris-Saclay University, CNRS UMR 9187, Inserm U1196, CEDEX F-91898, 91400 Orsay, France; 6 Breast, Gynecology and Reconstructive Surgery Unit, Curie Institute, 75005 Paris, France; 7 School of Medicine, Paris Cité University, 75006 Paris, France; 8 General and Digestive Surgery Department, Son Espases University Hospital, 07009 Palma de Mallorca, Spain; 9 HPB and Oncological Surgery Department, Virgen Macarena University Hospital, 41009 Seville, Spain

**Keywords:** low-grade ovarian cancer, lymphadenectomy, overall survival, period-free disease

## Abstract

**Simple Summary:**

Ovarian cancer ranks as the eighth most prevalent malignancy and the eighth deadliest among women. Histologically, epithelial ovarian cancers comprise 90% of all ovarian cancers, with serous ovarian cancer being the most prevalent subtype. Serous ovarian cancer is further classified into high-grade serous ovarian cancer and low-grade serous ovarian cancer. The latter is typically diagnosed at a younger age and carries a more favorable prognosis. However, it displays relative chemoresistance and represents a rare form of malignant ovarian tumor. Clinical guidance for patients with this tumor primarily relies on retrospective studies and subgroup analyses from ovarian cancer clinical trials. The actual impact, in terms of overall survival and disease-free intervals, of systematic lymphadenectomy in patients with low-grade ovarian cancer remains uncertain. Our present meta-analysis aims to explore the current knowledge in this area and assess the available evidence.

**Abstract:**

Objective: To evaluate the role of systematic lymphadenectomy in low-grade serous ovarian cancer (LGSOC) and determine its impact on clinical outcomes in overall survival (OS) and disease-free survival (DFS) terms. Methods: A comprehensive, systematic computer literature search on PubMed was performed using the following Medical Subject Headings (MeSH) terms: “low grade serous ovarian cancer” AND/OR “lymphadenectomy” AND/OR “staging” AND/OR “ovarian cancer” AND/OR “cytoreduction”. Separate searches were performed with MeSH terms on MEDLINE and EMBASE to extract all the relevant literature available. We included only patients with histologically confirmed LGSOC. Results: Three studies were considered in the quantitative analysis. Systematic lymphadenectomy in LGSOC failed to provide a significant OS or PFS benefit in LGSOC when compared to no lymphadenectomy in the entire (all the stages) population (for OS: HR = 1.15, 95% CI [0.42, 3.18] I2 = 84% and for PFS: HR = 1.46, 95% CI [0.63, 3.41], I2 = 71%), nor did it in the subtype analysis regarding FIGO stages. For FIGO early-stage I-II LGSOC, the DFS data were pooled (HR = 1.48, 95% CI [0.58, 3.78], I2 = 75%). In patients with advanced-stage (FIGO II–IV), we also failed to prove survival benefit for lymphadenectomy in OS (HR = 1.74, 95% CI [0.87, 3.48], I2 = 11%) or DFS (HR = 1.48, 95% CI [0.58, 3.78], I2 = 75%) compared to no lymphadenectomy. Conclusion: More extensive prospective research is mandatory to understand the real impact of lymphadenectomy on survival in LGSOC. The existing literature does not provide strong evidence.

## 1. Introduction

Ovarian cancer is a significant global health concern, with a high mortality rate among women. Transitioning economies, including European and North American countries, continue to have the highest incidence of ovarian cancer, while African countries tend to have among the lowest rates. Conversely, ovarian cancer mortality rates exhibit much less variability worldwide, partly due to the relatively poor prognosis associated with ovarian cancer in transitioning economies. Most women are diagnosed with advanced-stage cancer with a poor prognosis, partly driven by delays in diagnosis and unequal access to quality care [1].

Low-grade serous ovarian cancer (LGSOC) is a rare subtype of this malignancy, accounting for only 6–10% of epithelial ovarian cancers. Histologically, LGSOC presents as a uniform proliferation of cuboidal, low columnar cells, exhibiting mild-to-moderate atypia without nuclear pleomorphism. The mitotic index can reach up to 12 mitoses per 10 high-power fields, accompanied by a characteristic destructive invasion. The majority of LGSOC cases are linked to serous borderline ovarian tumors exhibiting a micropapillary/cribriform pattern, with the invasive component often featuring a micropapillary pattern. Moreover, estrogen receptors and progesterone receptors are expressed more frequently in LGSOC compared to HGSOC. Discrepancies in the reported positivity rates of steroid receptors are primarily attributed to variations in immunohistochemical analytic platforms and the thresholds used for defining positivity [2].

LGSOC is typically diagnosed at a younger age, has less aggressive behavior, and is associated with a better prognosis than HGSOC [3], but it is relatively chemo-resistant [4]. For that, primary maximal cytoreductive surgery [5,6,7], including total hysterectomy, bilateral salpingo-oophorectomy, omentectomy, and resection of macroscopic carcinomatosis lesion [8,9,10], is the cornerstone of the treatment and it is essential for the clinical prognosis of patients with advanced LGSOC [11].

Currently, there are various systemic therapeutic options, which include systemic carboplatin and paclitaxel, either as standalone treatments or in combination with endocrine agents for maintenance therapy. Endocrine therapy alone, typically with an aromatase inhibitor, is also a considered approach. Presently, national guidelines and standards of practice exhibit wide variation, and individual treatment teams’ preferences and perspectives often influence patients’ treatment paths without sufficient evidence. The real situation is that the benefit of traditional adjuvant systemic treatment as chemotherapy or hormonal treatment is unclear [12]. There are limited studies about classic adjuvant treatment in LGSOC, and these do not clarify the survival benefit of chemotherapy or hormonal treatment. Target therapies such as MEK inhibitors have been introduced in clinical practice [4]. MEK inhibitors have demonstrated significant promise, challenges related to funding, availability, and licensing pose substantial obstacles to their widespread integration into routine clinical care. Combining these drugs with other targeted therapies, such as BRAF inhibitors, shows potential in the treatment of LGSOC [13,14].

Although the Observatoire des Tumeurs Malignes Rares Gynécologiques (TMRG) recommends pelvic and para-aortic lymphadenectomy during surgery with a stated purpose, the survival benefit of lymphadenectomy in LGSOC remains unclear in the literature, mainly now that the LION trial has changed practice regarding lymphadenectomy in advanced ovarian cancer due to a lack of evidence of survival benefit [15]. Clinical guidance for patients with LGSOC is mainly based on retrospective studies and subgroup analyses of ovarian cancer clinical trials [16]. According to previous studies, lymph node metastasis occurs in around 70% of LGSOC patients, and this proportion increases with the International Federation of Gynecology and Obstetrics (FIGO) stage [17]. However, the clinical benefits of lymphadenectomy during surgery for LGSOC remain inconclusive [15]. Thus, it is essential to investigate the potential benefits and risks of lymphadenectomy during surgery in patients with LGSOC to optimize clinical management and improve patient outcomes.

In this study, we performed a literature review and subsequently a meta-analysis to evaluate the role of systematic lymphadenectomy in advanced low-grade serous ovarian cancer (LGSOC) and determine its impact on clinical outcomes. The study will contribute to assessing the prevalence of lymph node involvement in LGSOC and provide evidence-based recommendations on the indications for lymphadenectomy. The findings could inform clinical practice guidelines and improve the management and outcomes of patients with LGSOC.

## 2. Materials and Methods

### 2.1. Data Sources and Study Selection

A thorough and systematic computerized literature search was conducted on PubMed utilizing the following Medical Subject Headings (MeSH) terms: “low grade serous ovarian cancer” AND/OR “lymphadenectomy” AND/OR “staging” AND/OR “ovarian cancer” AND/OR “cytoreduction”. Separate searches were conducted using MeSH terms on both MEDLINE and EMBASE databases to gather all pertinent research available in the literature.

### 2.2. Study Selection

**Figure 1:**
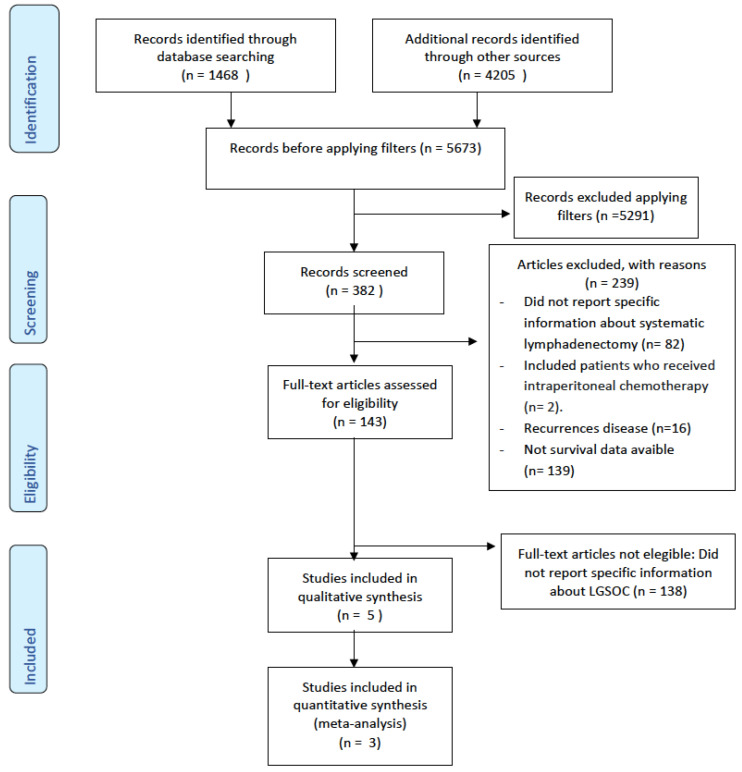
PRISMA [18] flow diagram.

Inclusion criteria were studies published in humans in English between January 2000 and December 2022. All forms of meta-analyses, prospective and retrospective studies, along with systematic reviews, were considered for inclusion in the present review. Two authors independently assessed the eligibility of all identified studies (Figure 1).

The study population comprised individuals diagnosed with histologically confirmed low-grade serous ovarian cancer (LGSOC).

We integrated studies that registered data about nodal staging with systematic lymphadenectomy and survival.

We systematically excluded studies that did not meet the following criteria:

Lack of specific information on systematic lymphadenectomy.

Absence of survival data.

Inclusion of cases involving recurrent disease.

Enrollment of patients who underwent intraperitoneal chemotherapy.

Non-consideration of grey literature; case reports, letters, and comments were expressly excluded from our analysis.

Our systematic review was submitted to the PROSPERO register and published in the database with the registration number: 403013.

### 2.3. Statistical Analysis

Age and follow-up time were presented as either mean or median, depending on the characteristics of each study. Survival outcomes were articulated through median time to recurrence/death, percentage of patients, disease-free survival (DFS), overall survival (OS) at 2, 3, or 5 years, and hazard ratios (HRs) or odds ratios (ORs) for DFS and/or OS. The diversity in survival metrics posed a challenge in data analysis due to its heterogeneity.

**Figure 2:**
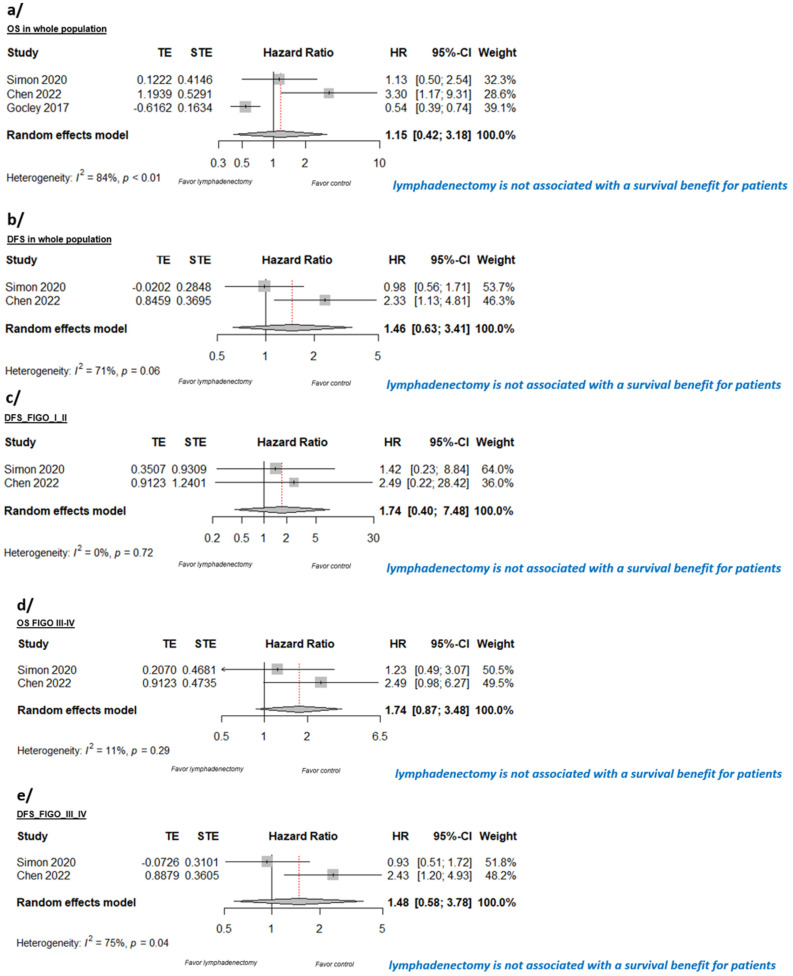
Meta–analysis for (**a**) OS in whole LGSOC population regarding systematic lymphadenectomy, (**b**) DFS in whole LGSOC population regarding systematic lymphadenectomy, (**c**) DFS in early-stage FIGO I–II LGSOC regarding systematic lymphadenectomy, (**d**) OS in advanced-stage FIGO III–IV LGSOC regarding systematic lymphadenectomy, and (**e**) for DFS in advanced-stage FIGO III–IV LGSOC regarding systematic lymphadenectomy. References: [19,20,21].

After the study selection and application of the exclusion criteria, we stratified studies included in the meta-analysis to systemic lymphadenectomy versus no systemic lymphadenectomy. We performed a global analysis (whole LGSOC population, Figure 2a,b) and then also a subgroup analysis: for the early-stage FIGO I-II (Figure 2c) and for advanced-stage FIGO III–IV separately (Figure 2d,e). Unfortunately, as we did not have a finite confidence interval for the HR of the OS for the early-stage group studied by Chen et al. [19], we could not carry out the meta-analysis for the OS concerning the early-stage Figo I II. Indeed, we only had one confidence interval for the HR, that given by Simon et al. [20].

In this work we used the meta packages available in the release 4.2.2 of the R software. In all the analyses, we computed hazard ratios (HRs) and then pooled the HRs either for OS and DFS using the random effects model to account for heterogeneity. The confidence intervals were considered two-sided and computed with a confidence of 95%. We applied the inverse variance method for which the weight is the inverse of the variance. Finally, we used the chi-squared test and the Cochrane Q-test to quantify the heterogeneity across studies, computing I2 for each endpoint and the Sidik–Johnkman estimator for tau.

### 2.4. Quality Assessment of Studies

The methodological quality of cohort studies was evaluated using the “Quality Assessment Tools of the National Heart, Lung, and Blood Institute” (NHLBI) [22].

## 3. Results

### 3.1. Evidence Acquisition

A literature search using MeSH terms yielded 5673 articles. After excluding grey literature such as case reports, letters, comments, and non-English papers, 382 records remained. Following abstract screening, 239 articles were excluded based on the aforementioned criteria. A total of 143 full articles were assessed for eligibility, and only 5 studies met the criteria for inclusion in the qualitative analysis (see Figure 1). The remaining 138 articles did not contain specific information regarding low-grade serous ovarian cancer (LGSOC).

### 3.2. Study Characteristics

In the five studies eligible for inclusion, two concerned patients in all the stages [19,20], two concerned only patients with advanced LGSOC [21,23], and one concerned only patients with early LGSOC [24].

Except one study (Nasioudis et al.) [23] where patients’ data were prospectively collected from participating commission-accredited cancer programs, all the other studies were of a retrospective nature.

The baseline characteristics of the five included studies are shown in Table 1.

Overall, 971 patients were included from all studies.

### 3.3. Qualitative Synthesis

Five studies were included for qualitative analysis. Four of them compared lymphadenectomy versus no lymphadenectomy [19,20,21,23], while one of them compared three different approaches to lymphadenectomy [24].

Similarly, four studies provided overall survival data [19,20,21,23], but only two of them provided disease-free survival data [19,20]. One of them presented survival data in a different format (odds ratio) [24]. Below, we will report in more detail the five studies that were ultimately included in the present meta-analysis.

Simon et al. [20] conducted a comparative study between two patient groups: one undergoing lymph node dissection and the other without this procedure. All cases of low-grade serous ovarian carcinoma (LGSOC) were rigorously confirmed through pathological review and subsequently treated with either primary debulking surgery (PDS) or interval debulking surgery following neoadjuvant chemotherapy (NACT-IDS). The study’s findings notably did not demonstrate any improvements in overall survival or progression-free survival (PFS) for patients with LGSOC who underwent systematic lymphadenectomy. The study also provided analysis in subgroups regarding FIGO stage or timing of the surgery (PDS or NACT-IDS), and they did not find a survival benefit for lymphadenectomy in any group. However, it is crucial to address a significant bias in this study—the fact that the group without lymphadenectomy had a 71% rate of CC-0/1 (optimal debulking), while the lymphadenectomy group had a much higher rate of 95.5% CC-0/1. This substantial bias profoundly impacts the outcomes of both overall survival (OS) and disease-free survival (DFS).

Chen Z. et al. [19] analyzed a database of 155 patients and employed propensity score matching (PSM) to mitigate potential biases when comparing patients who underwent lymphadenectomy to those who did not. All patients had a confirmed pathological diagnosis of low-grade serous ovarian carcinoma (LGSOC) and underwent surgery as their initial treatment. We utilized the study data following PSM to include individuals with minimized biases, resulting in the analysis of 78 patients. In this study, significant statistical differences were observed in favor of lymphadenectomy concerning disease-free survival (DFS) and overall survival (OS). Also, in this study, subgroup analysis was performed regarding FIGO stage. This allowed us to include Simon et al. [20] and Chen Z et al.’s [19] studies for our meta-analysis in a subgroup stratified for FIGO stage analysis and a comparison study that investigated the survival significance of lymphadenectomy in a rare histologic subtype of ovarian cancer. This study involved 281 patients (90 patients, specifically the LGSOC subtype). The patients were divided into two groups: one group underwent lymphadenectomy, while the other did not. All patients in the study presented with advanced-stage disease (stage III–IV), underwent primary debulking surgery with complete gross resection, and received adjuvant chemotherapy within 6 months. The study did not report on disease-free survival (DFS) in the global population of the study. For the LGSOC group, 33.3% of the patients underwent comprehensive lymphadenectomy. 66.7% of the lymph nodes removed had metastasis. Lymphadenectomy was not associated with an overall survival benefit in LGSOC patients (*p* = 0.82). The study did not provide a finite HR, so we could not include these survival data in our quantitative analysis (meta-analysis)

Gockley et al. [21] employed a database comprising 755 patients with low-grade serous ovarian carcinoma (LGSOC) and applied propensity score matching (PSM), resulting in a comparison of two groups, each consisting of 202 patients: one group underwent lymph node dissection, and the other did not. All patients in the study presented with advanced-stage disease (IIIC and IV) LGSOC. Patients who received neoadjuvant therapy were excluded. Once again, to minimize biases and ensure consistency in the meta-analysis, our study utilized the data obtained through PSM. The study did not provide any information regarding disease-free survival (DFS), so only overall survival data could be used in our quantitative analysis.

Chen J. et al. [24] conducted a study involving 59 patients with LGSOC in early-stage diseases, defined as FIGO stages I to IIa. The patients were divided into three groups. Group 1 involved no LN dissection or LN sampling, which meant the removal of none or only a few lymph nodes (less than ten pelvic LNs). Group 2 consisted of pelvic lymphadenectomy, which entailed removing more than 10 pelvic lymph nodes. Group 3 underwent sentinel lymph node dissection (SLND), involving the removal of more than 10 pelvic lymph nodes and five para-aortic lymph nodes.

To standardize the analysis, Group 1 was considered “no lymphadenectomy”, while Groups 2 and 3 were categorized as “lymphadenectomy”. This study showed a benefit in progression-free survival for the patients who underwent lymphadenectomy, but as this study did not provide hazard ratio data for disease-free survival (DFS) and overall survival (OS), we could not use it in our quantitative analysis.

The studies and their survival data are presented in Table 2.

Finally, we could only include three studies in the quantitative analysis where the HRs for survival were given [19,20,21]. The two other studies did not provide HRs specific to LGSOC nor allow their computation [23,24].

### 3.4. Quantitative Synthesis

The median follow-up for the patients who were included in the present meta-analysis was 56.56 months.

The median for OS or DFS in months was not computed due to the heterogeneity in the description of survival in the different studies.

Forest plots summarizing the difference in survival between patients who underwent systematic lymphadenectomy and those who did not are show in Figure 2. The pooled HR analyses did not reveal any statistically significant DFS or OS benefit associated with systematic lymphadenectomy in LGSOC in the whole population (all stages): for OS, pooled HR = 1.15, 95% CI [0.42, 3.18], I2 = 84% (Figure 2a); for DFS, pooled HR = 1.46, 95% CI [0.63, 3.41]), I2 = 71% (Figure 2b).

Moreover, when we analyzed the subtype of patients with stage I-II LGSOC, systematic lymphadenectomy failed to significantly improve DFS when compared to no lymphadenectomy (pooled HR = 1.74, 95% CI [0.40, 7.48], I2 = 0%, Figure 2c). Data of OS in early I-II stages with a HR and IC were only provided in one study, so we could not analyze it in the quantitative analysis. For patients with LGSOC in stages III-IV, systematic lymphadenectomy also failed to significantly improve OS (HR = 1.74; 95% CI [0.87, 3.48], I2 = 11% Figure 2d) or DFS (HR = 1.48; 95% CI [0.58, 3.78], I2 = 75%; Figure 2e) when compared to no lymphadenectomy in our analysis.

### 3.5. Quality Assessments

The quality of the five retrospective studies was assessed using the NHLBI study quality assessment tool [22] and was rated as “fair” in all cases (Table 3).

The most common biases were that the outcome assessors were not blinded to the exposure status of participants, the potential confounding variables for sample size justification were not measured or adjusted, and the measurement of confounding variables was missed.

## 4. Discussion

To our knowledge, this is the first comprehensive systematic review and meta-analysis in the literature designed to assess the OS and DFS benefits of systematic lymphadenectomy, specifically in the LGSOC subtype. We focused on patients diagnosed with LGSOC and aimed to elucidate the potential benefits of systematic lymphadenectomy. Our study involved a meticulous evaluation aimed at assessing the impact of lymphadenectomy on both overall survival (OS) and disease-free survival (DFS) in this specific patient population. To ensure the rigor and reliability of our findings, we adhered to stringent criteria, resulting in the inclusion of only five studies that met all these predefined criteria.

Finally, our analysis did not demonstrate a survival benefit of lymphadenectomy globally in patients with LGSOC nor in the sub-analysis regarding FIGO stages (early I–II FIGO and advanced III–IV FIGO stages).

Lymphadenectomy has garnered considerably more attention in the context of HGSOC [25,26,27] when compared to its application in LGSOC, primarily owing to the lower incidence of the latter. The seminal LION study [15] marked a pivotal moment in the field by leading to the abandonment of systematic lymphadenectomy in advanced epithelial ovarian cancer of FIGO stage IIB through IV. But in this randomized trial, only 4.5% of the tumors were the LGSOC subtype. However, it is important to note that this change in practice also affected LGSOC patients, in whom surgery is the cornerstone of their treatment.

The exclusion of low-grade ovarian cancer (LGSOC) patients in the “LOVE Study” [28] further emphasizes the differential focus on high-grade ovarian cancer (HGSOC) in research related to lymphadenectomy. It is an ongoing multicenter, randomized controlled, phase III trial whose objective is to evaluate the efficacy and safety of cytoreduction surgery with or without lymphadenectomy in stage IA-IIB epithelial ovarian and fallopian tube cancer. The hypothesis is that the oncological outcomes provided by staging surgery without lymphadenectomy are non-inferior to those of conventional completion staging surgery in these patients who have indications for post-operative adjuvant chemotherapy. This exclusion highlights the need for specific investigations into the role of lymphadenectomy in LGSOC cases, as the available evidence and clinical guidelines may not be directly applicable to this subgroup of patients. Despite the lack of specific evidence on LGSOC and its exclusion from studies like the LOVE study, protocols for LGSOC have been influenced and shaped by research on HGSOC. This meta-analysis aims to establish management strategies for LGSOC based on its unique evidence profile.

The involvement of lymph nodes in patients with LGSOC occurs in up to 70% of cases [17], with the para-aortic and pelvic regions the most affected, especially the right obturator fossa followed by the left obturator fossa. Lymphadenectomy is a procedure with secondary surgical risks and morbidity, mainly lymphatic complications [29]. Consequently, lymphadenectomy is a procedure that carries a substantial treatment burden, and the surgeon’s decision regarding its implementation may be influenced not only by disease characteristics such as stage or histology but also by the patient’s age, performance status, and concurrent medical conditions. Therefore, finding the balance between surgical risk and benefit in survival or as a staging technique is essential to know the best medical proposal to give to our patients.

These factors introduce numerous biases in retrospective studies, as patients with a poorer performance status tend to be categorized into the no-lymphadenectomy group, while younger and healthier patients are more likely to undergo lymphadenectomy. Accounting for such biases is a challenging endeavor, even when surgeons are cognizant of the inherent limitations of retrospective analyses, given all the available evidence to date.

While the overall trend in the data appears to fail to show the benefit of lymphadenectomy in terms of survival, it is crucial to address certain limitations inherent to our analysis. There are important limitations that prevent us from drawing definitive conclusions regarding the absence of a significant benefit associated with lymphadenectomy at this juncture. Only a few studies are available, and the heterogeneity among the studies limits the possibility of the inclusion of the studies in the final meta-analysis.

The relatively small sample size and the fact that the confidence interval crosses the threshold of 1 in the forest plot for hazard ratio (HR) raise concerns.

It emphasizes the need for larger, well-designed studies to provide more definitive insights into this critical aspect of ovarian cancer management.

The strength of our work is that it is the first meta-analysis in the literature about the OS and DFS benefits of systematic lymphadenectomy specifically in the LGSOC subtype. Additionally, we provided a subgroup analysis focused on patients in different stages of the disease. Notably, the results from this subgroup analysis yielded outcomes consistent with the overall analysis. However, it is important to reiterate the caution required in interpreting these results, given the limitations outlined earlier.

Although our study fails to demonstrate a survival benefit of lymphadenectomy for patients with LGSOC, it is important to highlight that the role of lymphadenectomy as staging is still valid, mainly in the early stages. An upstaging secondary to lymphadenectomy allows patients with initially early stages access to target therapy (to bevacizumab in this case) [30].

## 5. Conclusions

In conclusion, our meta-analysis highlights the necessity for more extensive, prospective research to truly comprehend the potential advantages of lymphadenectomy in low-grade ovarian cancer. With the limited number of studies available, all of which are retrospective, the need for larger, well-designed prospective studies becomes evident. The current evidence lacks a definitive answer regarding its utility, underscoring the imperative for further research to elucidate its benefits and risks across diverse clinical contexts.

## Figures and Tables

**Table 1:** Summary of the baseline characteristics of the five included studies.

Study (Year)	Design	Setting	Arms	Follow-Up (Months)	Patients (*n*)	Population	Age (Years)
Simon V. (2020) [20]	Retrospective cohort	TMRG Database	Lymphadenectomy vs. no lymphadenectomy.	Median: 27.5	126	LGSOC	53 (19–82)
Chen Z. (2022) [19]	Retrospective	Multiple medical centers	Lymphadenectomy, no lymphadenectomy.	Varied	155 78 (PSM)	LGSOC	47 (21–79)
Nasioudis D. (2021) [23]	Retrospective	National Cancer Database	Comprehensive lymphadenectomy, no comprehensive lymphadenectomy.	Median of 46.3 for no lymphadenectomy; median of 53.75 for those with lymphadenectomy.	90 LGSOC	Stage III–IV clear-cell, endometrioid, mucinous, and low-grade serous carcinoma patients	57 (17–89)
Gockley (2017) [21]	Retrospective PSM	National Cancer Database	Low-grade serous ovarian cancer, high-grade serous ovarian cancer; lymph vs no lymph from PSM.	Median: 72.7	755 LGSOC 404 (PSM)	Women 18 years and older diagnosed with advanced-stage (IIIC and IV) serous ovarian carcinoma	53.6 ± 15.34
Chen J. (2021) [24]	Retrospective	Single center	Three types of Lymph	Median: 85.2	59 LGSOC	LGEOC—LG-SOC, LG-MOC, or LG-EOC	-

PSM: propensity score match.

**Table 2:** Summary of the qualitative characteristics of the five included studies.

Study, Date	Control Arm	Intervention Arm	Primary Endpoint	Median DFS (Mo)	HR	95%CI	Median OS (Mo)	HR	95%CI	Other OS Data
Simon V. (2020) [20]	No lymphadenectomy (31)	Lymphadenectomy (91)	Overall survival (OS) and progression-free survival (PFS)	41	0.98	[0.56; 1.71]	130	1.13	[0.50; 2.54]	
Chen Z. (2022) [19]	No lymphadenectomy (39)	Lymphadenectomy (39)	Prognostic value of lymphadenectomy in patients with LGSOC	65	2.33	[1.13; 4.81]	90	3.30	[1.17; 9.31]	
Nasioudis D. (2021) [23]	No lymphadenectomy (60)	Comprehensive lymphadenectomy (30)	The impact of comprehensive lymphadenectomy on the survival of patients with advanced-stage epithelial ovarian carcinoma	-						90.32 mo *
Gockley (2017) [21]	No lymphadenectomy (202)	Lymphadenectomy (202)	The identification of factors associated with survival among patients with advanced-stage low-grade serous ovarian cancer	-	-	-	106.5	0.54	[0.39; 0.74]	
Chen J. (2021) [24]	No lymphadenectomy (22)	Lymphadenectomy (37)	Disease-specific overall survival (OS)							5y-OS * 82%

* HR is not shown.

**Table 3:** Quality assessments.

Study, Year	Criteria													
	1	2	3	4	5	6	7	8	9	10	11	12	13	14
Simon, 2020 [20]					NA			NA		NA				
Chen, 2022 [19]					NA			NA		NA				
Gokley, 2017 [21]					NA			NA		NA				
Nasioudis, 2021 [23]					NA			NA		NA				
Chen, 2021 [24]					NA			NA		NA				

1. Was the research question or objective in this paper clearly stated? 2. Was the study population clearly specified and defined? 3. Was the participation rate of eligible persons at least 50%? 4. Were all the subjects selected or recruited from the same or similar populations (including the same period)? Were inclusion and exclusion criteria for being in the study prespecified and applied uniformly to all participants? 5. Were sample size justification, power description, or variance and effect estimates provided? 6. For the analyses in this paper, were the exposure(s) of interest measured prior to the outcome(s) being measured? 7. Was the timeframe sufficient so that one could reasonably expect to observe an association between exposure and outcome if it existed? 8. For exposures that can vary in amount or level, did the study examine different levels of exposure as related to the outcome (e.g., categories of exposure, or exposure measured as a continuous variable)? 9. Were the exposure measures (independent variables) clearly defined, valid, reliable, and implemented consistently across all study participants? 10. Was the exposure(s) assessed more than once over time? 11. Were the outcome measures (dependent variables) clearly defined, valid, reliable, and implemented consistently across all study participants? 12. Were the outcome assessors blinded to the exposure status of participants? 13. Was loss to follow-up after baseline 20% or less? 14. Were key potential confounding variables measured and adjusted statistically for their impact on the relationship between exposure(s) and outcome(s)? Legend: yes: ■; no: ■; not applicable: NA.

## Data Availability

Data are contained within the article.
